# The effect of previous team environment on the current motivation in Japanese varsity soccer players

**DOI:** 10.3389/fspor.2026.1872084

**Published:** 2026-07-03

**Authors:** Yuto Yasuda, Takeshi Hamamura, Kazuki Inagaki, Ken Kato, Masaaki Koido, Hironobu Tsuchiya

**Affiliations:** 1 School of Social and Health Sciences, James Cook University Singapore, Singapore, Singapore; 2 Curtin School of Population Health, Curtin University, Perth, Australia; 3 Institute of Health and Sport Sciences, University of Tsukuba, Tsukuba, Japan; 4 School of Sport Sciences, Osaka University of Health and Sport Sciences, Sennan District, Japan

**Keywords:** collective activity hypothesis, past environment, self-enhancement motivation, socio-ecologic framework, varsity soccer

## Abstract

**Introduction:**

Sport psychologists have investigated the effect of team environments on athletes' performance and mental health, revealing that team environments played salient roles in forming athletes' psychological processes. Currently, the scholars' interests are towards the effect of the current team environments the athletes are immersed in, and the effect of their past has not gained as much attention. Given that research on social psychology in sports settings has been built on the premise that the athletes' minds are shaped through interaction with their environment, the systematic differences across the past team environments should also impact the athletes' psychological processes. As such, we examined how past team environments affect varsity soccer players' motivation by comparing players from public schools, private schools, and club teams in high school.

**Methods:**

In the current study, 159 varsity soccer players (86 from public schools, 45 from private schools, and 28 from club teams) completed the questionnaires to measure the degree of resources, performance emphasis within a team, intra-team competition, and self-enhancement motivation, in which players perceive themselves positively.

**Results:**

The results demonstrated that club teams showed the highest degree of resources, followed by private school teams, then public school soccer teams. The higher degree of resources was related to the emphasis on the enhancement of individual performance within a team. This emphasis was further associated with intra-team competition, which was eventually related to self-enhancement motivation.

**Discussion:**

The results imply the significance of past team environments on athletes. To deepen the understanding of psychological processes in athletes, the effect of other past team environments in which they were previously immersed is suggested to be further explored.

## 1 Introduction

Under the purpose of fostering healthy and effective environments for athletes, sport psychologists have examined the effect of team environments on athletes' psychological processes (1). Among the various psychological processes to be targeted, motivation has been heavily examined in the relationship with team environments, as motivation is associated with players' persistence and effort among other factors, both of which strongly impact their performance (2, 3). Indeed, empirical research has shown that coaching styles, team climate, and peer support all influence players' motivation (1). On the other hand, the scope of the research is limited to the effect of the “current” team environments on players' psychological processes e.g. (4), as sport psychologists' main interests are the enhancement of current performance from a psychological perspective. As such, they have focused on how to holistically support and optimize the surrounding environment in a given situation. However, in applied settings, coaches often check players' backgrounds to deepen their understanding of the players as well as the current situation. For example, soccer coaches often gain information about the teams the players previously belonged to for the understanding of the players. If this type of narrative in applied settings is scientifically valid, the past organizational environment may shape the athletes' motivation.

To fill in this gap between academia and applied settings, we examined the effect of the past team environment on team sport players' motivation by utilizing a theoretical framework in cultural psychology. A cultural psychology framework is well-suited to the scope of the current study, as cultural psychologists have investigated the effect of shared backgrounds in a culture on psychological processes. As such, the current study examined if and how the past team environment influences team sport players' motivation by drawing on the insights of cultural psychology.

### 1.1 Theoretical framework in cultural psychology: collective activity hypothesis

In cultural psychology, psychological patterns are assumed to be shaped through individuals' active engagement with a culturally different environment; culturally different characteristics of the environment create idiosyncratic patterns in human psychology (5). Following this assumption, cultural psychologists have examined the effect of ecological, institutional, and social environments (e.g., economic structure and social norms) on human minds across cultures (6, 7). Among the environmental characteristics examined in the literature, one of the established factors associated with psychological processes is the degree of collective activity. The previous literature asserts that the level of collective activity within a community systematically differentiates individuals' psychological processes, which is called the collective activity hypothesis (8). According to this hypothesis, collective activities involve a large number of group members, and people engage in cooperative behaviors and coordination among members. This high degree of collective activity fosters interdependence, while a lower degree of collective activity facilitates independence. One of the examples that corroborates this assertion is rice farming vs. wheat farming (9, 10). Rice farming requires cooperation, such as maintaining irrigation systems, and is labor-intensive for rice cultivation. This socio-ecological characteristic allows people in the rice-farming areas to get credence and construct a good reputation with others. On the other hand, wheat-farming does not require such collaboration, and people are relatively more independent in growing wheat, which fosters independence as a psychological characteristic. This systematic difference emerged even when rice-and wheat-farming are no longer the primary mode of subsistence (9, 10).

### 1.2 Motivational differences contingent with the degree of collective activity

Furthermore, individuals with independence and interdependence show their idiosyncratic patterns in motivation. On one hand, individuals with independence place emphasis on autonomy and controllability (5, 11, 12). With these emphases, those with independence strive to be self-reliant and actualize their personal goals (12). This self-reliance tendency is supported by self-enhancement motivation: by inflating positive aspects of self, they believe in their capability and self-sufficiency (13–15).

In contrast, motivation for self-enhancement is significantly attenuated in individuals with interdependence (13, 14, 16–19). Rather, they exhibit a self-improvement tendency, where they are attuned to the perspectives of others and assess themselves accordingly to maintain and improve how they are perceived (13, 14), which is rooted in the emphasis on social relationships and harmony (5, 12). Individuals with interdependence predominantly have self-improvement motivation to maintain their social relationships with significant others and fulfill their responsibilities within society: avoiding conflicts and considering which aspect of self to improve to achieve their roles within their society (12).

The collective activity hypothesis and the following motivational difference would fit well with the characteristics of team sports, specifically interactive sports such as soccer, rugby, and basketball. In interactive sports, players are required to collaborate to some extent to achieve optimal team performance. However, the degree of collective activity may systematically differ across teams depending on team characteristics. To examine this possibility, the current study focused on the sports environment in Japan, given that Japanese sports contexts show systematic variations in collective activities across different competitive environments.

### 1.3 Characteristics of the team environments in Japanese soccer

In Japan, sports activities are popular among Japanese students as schools offer extracurricular activities such as soccer, baseball, basketball, tennis, and volleyball, from which students choose one. Notably, more than 40% of high school students belong to a sports club (20). One of the most popular sports in high school is soccer. Currently, more than 150,000 high school students are registered as soccer players at the Japan Football Association (21). Reflecting this popularity, a well-organized soccer infrastructure has been established. There are various divisions in well-structured leagues, where promotion and relegation occur at the end of each season see (22). Additionally, several national high school tournaments are held annually and are broadcast nationally to gain public attention. By demonstrating their exceptional skills, players may also be scouted by professional teams. Within this well-structured soccer environment, Japanese students typically play for their school team, although the environment tends to differ markedly between public and private schools.

Alternatively, high school students can join a private club to play. Through tryouts and specialized programs, these clubs aim to identify and prepare the next generation of elite soccer players. These club teams belong to the same leagues as school teams; leagues for high school players bring together teams from public schools, private schools, and clubs altogether. The current study investigated whether the differences among these teams can be seen through the lens of the collective activity hypothesis, considering variations in terms of resource availability, emphasis on individual performance, and intra-team competition.

#### 1.3.1 The degree of collective activities of each team type

The nature of team sports is to play collaboratively and coordinate with teammates (23), so there should not be considerable differences in the emphasis placed on team performance within a team, regardless of the team type. However, there are reasons to think that the difference in the degree of performance enhancement of individual players is contingent on available resources. On the one hand, public schools are required to follow guidance from the government, so available resources at their discretion tend to be more limited at public schools, which may not allow the teams to focus on enhancing individual performance to compete against opposing teams. Instead, the teams may strive to increase the possibility of winning a competition by collaborating with each other with limited resources. This high collective activity may be associated with self-improvement tendency to fulfill their respective roles within the team.

In contrast, teams at private schools tend to have more resources available following the Private School Act, which allows private schools autonomy over their operations and management (24). For instance, teams at private schools may hire nutritionists and physiotherapists. Another example may be the quality of the facilities, such as the quality of the gym, field, and clubhouse. With these resources, teams may be able to focus on individual as well as team performance. As such, the emphasis on collaborative activities may be weaker than in public school teams. As private school teams are hypothesized to have fewer levels of collective activity, we hypothesized that they would show less self-improvement tendency compared with their counterparts in public schools.

Lastly, at club teams, their objective is to identify and foster as many talented players as possible to nurture the next generation of elite soccer players and potentially send them to professional teams (25), thereby enhancing the club's value. For this purpose, a stronger emphasis is placed on individual players' performance enhancement by investing substantial resources such as facilities and human resources (25). Thus, players at club teams would be immersed in an environment where individuals' performance is emphasized with plenty of resources. Furthermore, given the highly competitive nature of securing a professional soccer contract, intra-team competition may be intense. These characteristics may result in the lowest level of collaborative activity among the three team types. In this team environment, players may attempt to rely on themselves and focus on their own personal excellence, which is compatible with self-enhancement motivation.

### 1.4 The current study

**Figure 1:**
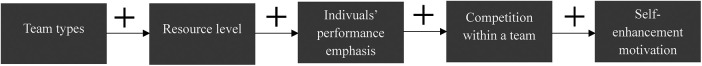
Hypothetical model in the relationship between team types, resource levels, competition within a team, and self-enhancement motivation. *Note. Team types were entered 1 (public schools), 2 (private schools), and 3 (club teams).

As discussed above, the current study examined whether and how the degree of collective activity that these players experienced at the high-school level is related to their motivation and tendency towards self-enhancement/self-improvement. To do so, we recruited university-level soccer players. Drawing on previous literature, we proposed a hypothetical model regarding the relationship between team types, resource levels, emphasis on individual performance, intra-team competition, and motivation (Figure 1).

In addition, self-enhancement motivation is more evident specifically when the situation diminishes individuals’ self-esteem, for example, through receiving negative feedback or experiencing failure as a means of self-protection (26, 27). Applying this reasoning to sports contexts, athletes may view themselves more positively, specifically after losing a competition, to compensate for the situation they are facing and maintain their self-esteem. In the current study, we measured self-enhancement motivation separately in the contexts of winning and losing a competition.

Lastly, even though the effect of socio-economic status (SES) is not the primary focus of the current study, we cannot and should not ignore its effect in our hypothetical model. Previous literature revealed that individuals with high SES tend to show independent characteristics while their counterparts with low SES tend to display interdependent tendencies due to the amount of resources available to them (28). As independence is closely aligned with the environmental characteristics of club teams, the relationship between the characteristics of club teams and self-enhancement motivation may be confounded by the effect of high SES. To determine whether the effect of athletes' backgrounds remains while controlling for the effect of SES, the current study included SES as a covariate.

## 2 Methods

### 2.1 Participants

Varsity male soccer players who competed in the first division of the Kanto League, one of the highest leagues in Japanese universities, participated in the current study. As the purpose of the study was to examine motivational variations among players who had played at public high schools, private high schools, and club teams, 5 data from participants who began playing soccer at university were excluded from the data analysis. Additionally, 7 data from the student manager, physical trainer, and coaching staff of the team were excluded. Before deleting incomplete data, we conducted an MCAR test, and the result was not significant, *χ*²(38) = 28.910, *p* = .856, denoting that missing data were at random. Following this result, 10 incomplete data were excluded. Subsequently, we targeted 159^1^ (*M*age = 20.47, *SD* = 1.39) for data analysis. The required sample size was calculated following Preacher and Coffman (30) with .05 for alpha, 20 degrees of freedom, .80 for power, .04 for null RMSEA, and .10 for alternative RMSEA, which resulted in a required sample size of 147. The values for null and alternative RMSEA were selected because the null value (.04) represents a stringent criterion for close model fit, following recommendations for stricter fit evaluation e.g. (31), whereas the alternative value (.10) represents poor model fit (32). The actual sample size exceeded the minimum required for conducting appropriate analyses. Of the 159 participants, 86 had played at public schools, 45 played at private schools, and 28 played at club teams. Ten played goalkeepers, 54 played defenders, 69 played midfielders, and 26 played strikers. Lastly, 42 were first-year students, 50 were second-year students, 26 were third-year students, and 41 were in their fourth-year or beyond.

### 2.2 Measures

#### 2.2.1 Ecological environment of the teams

The participants first indicated which type of teams they had played soccer at high school: public school, private school, or club team. Then, they rated the specific aspects of their team environment, as indicated below.

#### 2.2.2 Resource levels

Following Won and Chelladurai (33) and Xiang et al. (34), the substantiality of financial (e.g., budgets, sponsorship), human (e.g., nutritionists, physiotherapists), and environmental (e.g., facility, gym) resources was measured from 1 (not enough at all) to 7 (very abundant) with the anchors defined as “not enough at all” (no resources at all), “average” (equivalent to an average high school), and “very abundant” (equivalent to professional teams). Then, the mean scores of these three types of resources were calculated to produce the overall resource level. Higher scores indicate greater resources available for the teams. Cronbach's alpha was.87 in the current study.

#### 2.2.3 Emphasis on performance

The emphasis placed on individual and team performance within their teams was measured by two items: “How much did your team place emphasis on the enhancement of individual performance?” and “How much did your team place emphasis on the enhancement of team performance?” from 1 (not at all) to 7 (very much).

#### 2.2.4 Intra-team competition

The degree of intra-team competition was measured by one item: “How much did competitions within your team exist at your high school team?” from 1 (not at all) to 7 (very much).

#### 2.2.5 Self-enhancement motivation

Self-enhancement motivation is defined as the inflation of positive aspects of self to believe in their self-sufficiency and self-sustenance (13–15). This motivation becomes more salient in negative situations (26, 27). Following this definition and the characteristic, we assumed that self-enhancement motivation emerges when they reflect on their own performance after a competition. As such, the following instructions were developed.

“We will ask you how much you reflect on what went well and what did not go well for you in the competition immediately after it. Please respond while imagining a rival team whose performance level is similar to that of your own team.”

Then, the participants rated how much they would reflect on the positive and negative sides of their performance after winning and after losing a competition from 1 (do not reflect on it at all) to 7 (reflect on it very much). To measure self-enhancement motivation, the scores for reflection on negative sides of the performance were subtracted from the scores for positive sides of their performances, with higher scores indicating greater self-enhancement motivation. This subtraction procedure was implemented by Kitayama et al. (35) to compute the level of self-enhancement.

#### 2.2.6 Subjective socio-economic status

The participants' socio-economic status was measured by utilizing the ladder questionnaire created by Adler et al. (36). In this questionnaire, the participants saw a hypothetical ladder, imagining that a higher place on the ladder indicates a higher status in their society, and a lower place on the ladder indicates a lower societal status. Then, they were asked to indicate their position on the ladder, ranging from 1 (highest SES) to 10 (lowest SES). Constructive validity and reliability of this questionnaire have been demonstrated by Giatti et al. (37) and Ferreira et al. (38).

### 2.3 Procedures

The current study was approved by the ethics committee of the fourth and sixth authors' institution. A varsity soccer team was recruited to participate in the study. At the beginning of a team meeting, an online survey was distributed to the participants. The participants first read an informed consent form and proceeded to the main study upon providing consent. Then, they completed questions regarding the reflection on the positive and negative sides of themselves after winning and losing a competition. Subsequently, they answered questions regarding their soccer activities at high school, following demographic information (age, position, and university year).

## 3 Results

### 3.1 Descriptive analyses

Prior to the main analyses, the descriptive analyses were conducted. The means and standard deviations for each variable are presented in Table 1.

**Table 1:** Descriptive analysis.

Variables		Public schools		Private schools		Club teams	
		M	SD	M	SD	M	SD
Resources		3.24	1.16	4.10	1.33	5.44	1.33
Emphasis on individual performance		4.01	1.37	4.58	1.47	5.71	1.24
Emphasis on team performance		4.67	1.19	4.82	1.27	5.25	1.35
Intra-team competition		3.72	1.58	4.20	1.87	4.75	1.48
Self-enhancement motivation	After winning	1.21	2.02	1.22	1.51	1.21	1.81
	After losing	−1.78	1.86	−1.42	2.17	−1.57	1.83
SES		5.01	2.02	5.04	1.93	3.61	1.29

### 3.2 Correlation analysis

As shown in Table 2, team types were positively correlated with resource levels, emphasis on individual performance, team performance and intra-team competition (*r* = .161−.545, *p* = .043-<.001), while being negatively correlated with SES (*r* = −.225, *p* = .004). Resource levels were also positively related to emphasis on individual (*r* = .433, *p* < .001) and team performance (*r* = .354, *p* < .001), and intra-team competition (*r* = .492, *p* < .001), whereas being negatively related to SES (*r* = −.244, *p* = .002). Next, Emphasis on individual performance was positively related to emphasis on team performance (*r* = .237, *p* = .003) and intra-team competition (*r* = .369, *p* < .001) and negatively related to SES (*r* = −.259, *p* < .001). Emphasis on team performance was positively related to intra-team competition (*r* = .405, *p* < .001). Intra-team competition was positively related to self-enhancement motivation after losing (*r* = .236, *p* = .003) but negatively related to SES (*r* = −.165, *p* = .038). Lastly, self-enhancement after winning was positively correlated with their counterparts after losing (*r* = .216, *p* = .006).

**Table 2:** Correlation analysis.

Variables	1	2	3	4	5	6	7	8
1. Team type		.545***	.408***	.161*	.231**	.002	.059	−.225**
2. Resources			.433***	.354***	.492***	−.006	.088	−.244**
3. Emphasis on individual performance				.237**	.369***	.002	.057	−.259***
4. Emphasis on team performance					.405***	.017	−.056	−.126
5. Intra-team competition						.114	.236**	−.165*
6. Self-enhancement motivation after winning							.216**	.118
7. Self-enhancement motivation after losing								−.052
8. SES								
Skewness	.730	.106	−.041	−.256	.156	.372	.225	.547
Kurtosis	−.927	−.813	−.860	−.003	−.948	−.391	−.611	−.245

* *p* < .05.

** *p* < .01.

*** *p* < .001.

Team types were computed as followed: public school = 1, private school = 2, club team = 3. This table demonstrates the correlation between each variable.

### 3.3 Ecological factors across three team types

#### 3.3.1 Resource levels

To examine differences in the resource levels, a one-way ANCOVA was conducted with SES as a covariate. Levene's test showed that the assumption of homogeneity of variance is met, *F*(19, 136) = 0.707, *p* = .807. The results indicated a significant effect of team type, *F*(2, 155) = 29.30, *p* < .001, $\eta_{p}^{2\;}$ = .274. As expected, the subsequent Bonferroni-adjusted *post hoc* comparisons showed that the club teams had significantly more resources (*M* = 5.44, *SD* = 1.33) than private school teams (*M* = 4.08, *SD* = 1.33), *t*(71) = 4.25, *p* < .001, *d* = 1.02, which was significantly higher than public school teams (*M* = 3.24, *SD* = 1.16), *t*(129) = 3.74, *p* < .001, *d* = .69. The results supported our hypothesis regarding the degree of resources across the three types of soccer teams.

#### 3.3.2 Team and individual performance

To explore the emphasis on team performance and individual performance, a 3 (team type: public school vs. private school vs. club team)×2 (performance emphasis: team performance vs. individual performance) mixed factorial ANCOVA was conducted with SES as a covariate. Levene's test for team types showed that the assumption of homogeneity of variance is met, *F*(2, 156) = 1.753, *p* = .177 for individual performance, *F*(2, 156) = 0.018, *p* = .982 for team performance. The results revealed a significant main effect of team type *F*(2, 155) = 10.32, *p* < .001, $\eta_{p}^{2}\;$ = .117, but no significant main effect of performance emphasis, *F*(1, 155) = 0.254, *p* = .615, $\eta_{p}^{2}\;$ = .002. The interaction effect was also significant, *F*(2, 155) = 3.90, *p* = .022, $\eta_{p}^{2}\;$ = .048. The Bonferroni-adjusted *post hoc* comparisons revealed that the public school teams emphasized team performance (*M* = 4.67, *SD* = 1.19) significantly more than individual performance (*M* = 4.01, *SD* = 1.37), *t*(85) = 3.96, *p* = .001, *d* = 0.43. In contrast, there was no significant difference between individual and team performance in private school teams (individual performance: *M* = 4.58, *SD* = 1.47, team performance: *M* = 4.82, *SD* = 1.27), *t*(44) = 0.81, *p* = .328, *d* = 0.12 and club teams (individual performance: *M* = 5.71, *SD* = 1.24, team performance: *M* = 5.25, *SD* = 1.35), *t*(27) = 1.79, *p* = .144, *d* = 0.34. Furthermore, emphasis on individual performance was the highest in club teams (*M* = 5.71, *SD* = 1.24), followed by private school teams (*M* = 4.58, *SD* = 1.47), *t*(71) = 3.40, *p* = .080, *d* = 0.82, then public school teams (*M* = 4.01, *SD* = 1.37), *t*(129) = 2.19, *p* = .002, *d* = 0.40. Overall, team performance was consistently emphasized across all team types, while the degree of emphasis on individual performance varied between the three. Notably, as expected, teams at public schools emphasized team performance more than individual performance.

#### 3.3.3 Intra-team competition

To compare the degree of intra-team competition across the three groups, a one-way ANCOVA was conducted by controlling for the effect of SES. Levene's test showed that the assumption of homogeneity of variance is met, *F*(19, 136) = 0.816, *p* = .686. The results showed a significant effect of team type, *F*(2, 155) = 3.30, *p* = .040, $\eta_{p}^{2}$  = .041. As expected, the subsequent Bonferroni-adjusted *post hoc* comparisons showed that club teams reported a significantly higher level of intra-team competition (*M* = 4.75, *SD* = 1.48) than public school teams (*M* = 3.72, *SD* = 1.58), *t*(112) = 3.03, *p* = .014, *d* = .66. The score of private school teams (*M* = 4.20, *SD* = 1.87) did not differ significantly from public school teams or club teams. These results partially supported our hypothesis regarding the degree of intra-team competition levels across team types.

#### 3.3.4 Self-enhancement motivation

To examine the differences in self-enhancement motivation across competition outcomes and team types, a 3 (team type: public school vs. private school vs. club team)×2 (outcome: winning vs. losing) mixed factorial ANCOVA was conducted with SES as a covariate. Levene's test showed mixed results, *F*(2, 156) = 3.785, *p* = .025 for winning condition, *F*(2, 156) = 0.857, *p* = .426 for losing condition. Although group sizes were unequal and Levene's test was significant for the win condition, the actual degree of variance heterogeneity was minimal. The largest-to-smallest variance ratio was only 1.80 (4.07 for club teams, 2.27 for private schools), suggesting that the violation was unlikely to meaningfully affect the results. Therefore, the mixed ANOVA was retained. The results revealed a significant main effect of outcomes *F*(1, 155) = 16.19, *p* < .001, $\eta_{p}^{2}$ = .095. Specifically, players showed greater self-enhancement motivation after winning (*M* = 1.21, *SD* = 1.84) than after losing (*M* = −1.64, *SD* = 1.94). However, there was no significant main effect of team type, *F*(2, 155) = 0.27, *p* = .77, $\eta_{p}^{2}$ = .003 nor a significant interaction effect between team type and outcome, *F*(2, 155) = 0.37, *p* = .69, $\eta_{p}^{2}$ = .005. These results demonstrated that participants tended to show greater self-enhancement motivation after winning than after losing.

### 3.4 Structural equation model

To examine the hypothetical model, we conducted structural equation modeling to examine the relationships between these variables while controlling for the effect of SES. Based on previous research by Schermelleh-Engel and Moosbrugger (39), the indices of goodness-of-fit that were used in the current study were the Normed Fit Index (NFI; ≥.90 indicating good fit), the Comparative Fit Index (CFI; ≥.95 indicating good fit), and the Root Mean Square Error of Approximation (RMSEA; ≤.08 indicating good fit). Based on the modified indices, the error variables were connected between resource levels and intra-team competition. This is theoretically reasoned following the assertion of the collective activity hypothesis (8): in the environment with lower resources, players need to cooperate with each other to compete as a group, whereas players with higher resource availability may focus on their own status (e.g., starting member) rather than cooperating as a group. Multiple indicators were selected as no single index is considered a perfect measure of fit (29). The indices for the current model were NFI = .937, CFI = .983, and RMSEA = .046, which indicate a good fit for the model.

**Figure 2:**
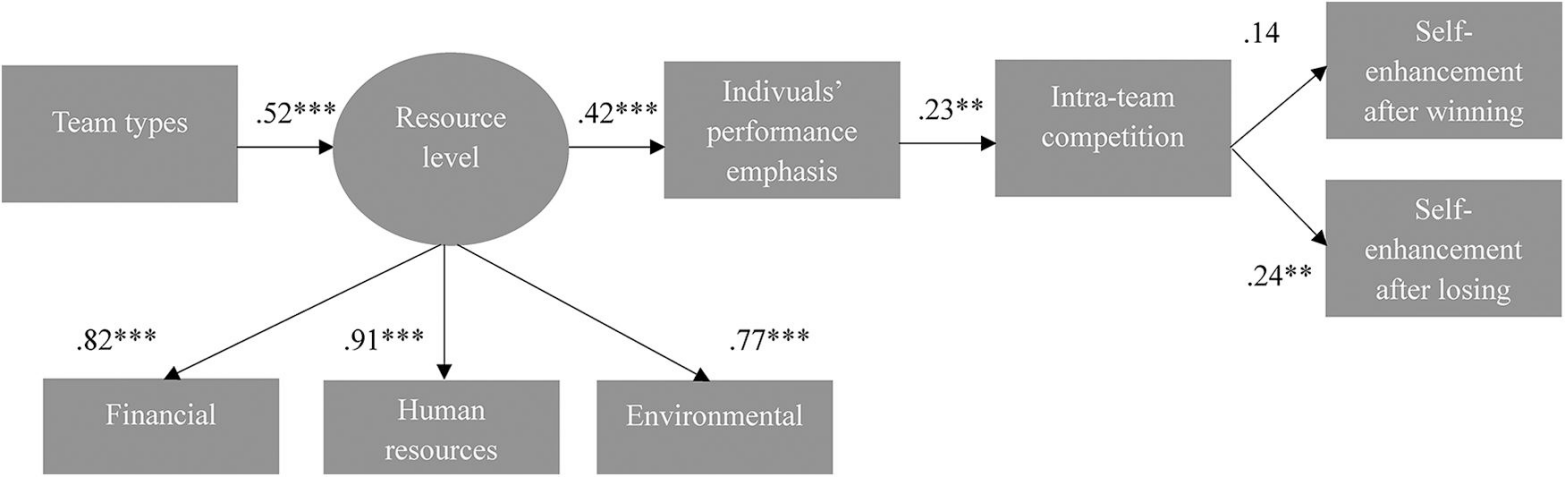
The results of structural equation modeling. *p < .05, ***p < .001 The following are the standardized coefficients from SES to each variable. SES->resources (*β* = −.14), SES->individuals’ performance emhapsis (*β* = −.15*), SES-> intra-team competition (B = −.11) SES->Self-enhancement after winning (*β* = −.14) SES->Self-enhancement after losing (*β* = −.14). The indirect effect from team types to self-enhancement motivation was significant after losing a competition (*β* = .029, *p* = .008, 95%IC[.010, .106]) and after winning a competition (*β* = .016, *p* = .045, 95%IC[.000, .081]).

As shown in Figure 2, the model revealed that resource levels were the highest in club teams, followed by private schools, and then public schools (*β* = .52, *p* < .001). Higher resource levels were associated with greater emphasis on individual performance (*β* = .42, *p* < .001), which was further related to higher levels of intra-team competition (*β* = .23, *p* = .003). Lastly, due to the systematic differences in backgrounds in the teams at high school, the degree of self-enhancement motivation was also varied, indicating that high intra-team competition was positively related to greater self-enhancement motivation; this relationship was specifically evident after losing a competition (*β* = .24, *p* = .003) compared to after winning a competition (*β* = .14, *p* = .083). The indirect effect from team types to self-enhancement motivation was also calculated by the bias-corrected percentile method, which was significant after losing a competition (*β* = .029, *p* = .008, 95%CI[.010, .106]) and after winning a competition (*β* = .016, *p* = .045, 95%CI[.000,.081]). These results indicated that the relationship between team types and self-enhancement motivation was mediated by resource levels, individual performance emphasis, and intra-team competition levels.

## 4 Discussion

The current study found that various soccer team types showed systematically different degrees of collective activity and, accordingly, different motivations. This supports the collective activity hypothesis in the athlete population. The following discussion elaborates on 1) the degree of self-enhancement between the three types of teams, 2) implications, and 3) limitations and future directions.

### 4.1 The degree of self-enhancement motivation among the three types of teams

The present study found that team types (public schools vs. private schools vs. club teams) were characterized by varying levels of resource substantiality, emphasis on individual performance, and intra-team competition, which further differentiated the degree of self-enhancement motivation. As hypothesized, the results indicated that club teams, private school teams, and public school teams showed the highest levels of substantiality of resources in descending order, which was similarly reflected in the emphasis on individual performance and intra-team competition. As discussed earlier, club teams tend to focus on individual performance and invest resources to maximize the number of players progressing to professional teams. Moreover, private schools can assign their resources at their discretion (24). On the other hand, public schools have limited available resources to do so as they are supposed to follow the guidance of the government. The results of the current study appear to reflect these contextual differences. Furthermore, greater resource availability may allow teams to dedicate attention to individual performance development. It is reasoned that teams with plenty of resources can afford to enhance individual performance as well as team performance, which further intensifies the intra-team competition to be selected as a starting member. This reasoning indicates that emphasis on individual performance and intra-team competition with a vast amount of resources disregards collective activities within a team in the Japanese context.

Moreover, these team differences were associated with the self-enhancement motivation. When the varsity players were in environments characterized by the emphasis on individual performance and high intra-team competition with plenty of resources, they were more likely to show self-enhancement motivation, where they reflected more on the positive than the negative side of their performance after a competition. This was specifically evident after losing a competition, which may reflect a defense response to the threat to their self-esteem (26, 27). Conversely, the results demonstrated that de-emphasis on individual performance and less intra-team competition with limited resources may be associated with less self-enhancement motivation. This relationship remained significant after controlling for the effect of SES. On one end, as a training institution for future professional players, club teams tend to spend plenty of resources with the purpose of maximizing the number of players progressing to professional soccer teams, with evidence of high emphasis on individual performance and intra-team competition. These idiosyncratic characteristics of club teams may implicitly encourage individual players to show individual excellence and rely on their performance, which is compatible with the self-enhancement motivation. On the opposite end, public school teams need to show team performance with limited resources. These environmental characteristics may result in the promotion of collective activities such as tactical, physical, and psychological strategies to win against stronger teams. Even team management tasks (e.g., scheduling, field settings) may be carried out collectively. These high levels of collective activity may diminish athletes' self-enhancement motivation and rather foster self-improvement motivation more. As hypothesized, private school teams fell between club teams and public school teams with regard to the degree of resources, emphasis on individual performance, and intra-team competition. With their discretion of the resources, they can allocate more resources to the team than public school teams do, but the resource level does not reach that of their counterparts in club teams. These moderate resource levels are associated with moderate levels of emphasis on individual performance and intra-team competition levels, which further assist in showing a relatively moderate level of self-enhancement motivation.

Overall, the results supported the application of the collective activity hypothesis within sports contexts. This hypothesis asserts that the degree of collective activities facilitates characteristics of interdependence (40). Indeed, the previous literature suggested that collective activities affect psychological processes such as the importance of reputation and the perceptions of shared emotions (8, 41). In alignment with this line of research, the current study identified the socio-ecological factors where the collective activity hypothesis is applied in the athletic contexts and how these factors affected the athletes' motivation. In this sense, the current study led to the confirmation and advancement of the collective activity hypothesis.

### 4.2 Implications

With the significant results, the present study encompasses several important academic and applied implications. Firstly, the current study has several academic implications. As aforementioned, even though the effects of social factors have received considerable attention to deepen the understanding of athletes' psychological processes, scholarly focus has remained largely limited to the social environments in which athletes are currently immersed at the time of study. Conversely, the effect of past environment has not gained as much attention from scholars. With the limited attention from scholars, the results of the current study indicate that the previous sports environments may play a pivotal role in shaping athletes' psychological processes, which will assist in completing the understanding of athletes' psychological processes. This is consistent with the premise and assumption in cultural psychology that the human mind is constructed through active interaction with one's environment (5). To further expand this line of research, it is suggested to explore the effects of previous sports environments on psychological processes beyond motivation such as cognition, emotions and even behavior.

From a practical point of view, the current study contributes to personalized sports psychology interventions. The current trend in sports psychology is to figure out psychologically universal processes and create one-size-fits-all interventions (42). Although the interventions in sports psychology showed certain effectiveness for performance enhancement (43), the nuanced variations in athletes' psychological processes have not gained sufficient attention from scholars (44). As the counterargument against this trend, the current study examined Japanese athletes and revealed that socio-ecological factors systematically affected athletes' motivation. The results of the current study imply that the process of performance enhancement may differ among players from various environmental backgrounds. For example, players from public schools may focus on identifying and improving their weaknesses, while those from club teams may strive to maintain and further enhance their strengths. Given that psychological interventions should be individually adjusted (45), the effect of socio-ecological factors which impact psychological processes should be continuously investigated.

### 4.3 Limitations and future directions

The current study encompasses significant implications, as discussed. However, the current study is not without limitations. Firstly, causal relations could not be identified. The research design of the current study was cross-sectional, whereby participants completed questionnaires at a single time point. As a result, the directionality of the observed relationships remains unclear. Future research is suggested to conduct empirical research that manipulates the available resources, the emphasis on individual performance and intra-team competition, and then examine whether the motivation systematically changes.

Next, we note the unequal participants across groups. Relatively, we have more participants from public schools and fewer participants from the club team because we only collected data from one university to minimize the potential confounding factors by collecting data across multiple universities. Club teams should be more competitive as they have selection for entry, whereas public schools are open to every interested player. Thus, this unequal assignment was reasonably predictable. Additionally, we examined the homogeneity of variance across three team types and confirmed that the variances in the three groups are similar. Nevertheless, this unequal sample size and relatively small sample size of club teams may affect statistical stability in group comparison (46) and SEM estimates (29). For example, with more participants from club teams, the significance levels in ANCOVA and the coefficients in SEM may fluctuate. To solve this potential issue, it is recommended to collect data from multiple universities while controlling for potential confounding factors. Related to the above limitation, there are potentially other confounding factors. We included SES as a confounding factor because SES is theoretically related to self-enhancement motivation (28). However, there may be other potential factors related to self-enhancement motivation such as perceived skill levels, injury history, and starting age of playing soccer. As aforementioned, we attempt to minimize potential confounding factors by collecting data from one university, but future research is suggested to include other potential confounding factors.

Another limitation of the present study is generalizability. The results of the current study will be applied to competitive male varsity soccer players in Japan, and the replication in female athletes, lower competitive levels, other sports, and other countries has not been guaranteed. For example, the current study did not include female participants as the leagues and structural environments for female players are not yet as well-established as those of their male counterparts. Thus, it would be difficult to collect data from female players across all three team types, which raises concerns about whether comparable differences in team environments across the three team types would be present. Nevertheless, previous research indicates that the degree of self-enhancement motivation is higher in men than in women (47), so it would be worth exploring how gender interacts with the current findings. Also, each country has a unique sports environment; therefore, it is uncertain whether similar differences in resource availability, performance emphasis and intra-team competition would be seen in other countries. Following this uncertainty, it is not ensured whether corresponding differences in motivation across team types would emerge in other cultural contexts. To explore whether the results of the current study are unique to the population of Japanese athletes or can be replicated in other countries, future research should conduct cross-cultural studies. Gender and cultural differences are potential factors which may cause different results from the current results. Future research should examine a different population to confirm the replicability of the current study.

Another limitation is that all variables were assessed using self-report questionnaires. Specifically, regarding question items about high school environment, they were retrospectively asked, and as such, the possibility of the hindsight effect (48) and the risk of response biases such as social desirability (49) or acquiescence biases (50) still remain. To minimize these effects, we gave a reference for resource levels by showing the definition of the level of resources. In this way, we attempted to make the scale have the same meaning across the participants. However, these effects may still have occurred in items for emphasis on team and individual performance and intra-team competition, which are difficult to give a reference or definition of the labels. Future research could strengthen the findings by incorporating multiple methods of assessment, such as objective and subjective measures.

Lastly, we need to note several ways to measure self-enhancement motivation. For example, Yasuda and Masuda (51) asked the participants to assess their positive and negative sides of themselves as an athlete and compared them to measure self-enhancement. The current study showed significant results when the participants reflected on their positive and negative performance in winning and losing games. Future research is suggested to replicate the results in different measures of self-enhancement motivation. Related to this point, some variables (e.g, individuals' performance emphasis, intra-team competition) were measured by a single item. Even though single-item questionnaires showed acceptable validity and reliability (52, 53), some literature is still concerned about the reliability of this, depending on the research questions and purposes (54, 55). Thus, it is suggested that future research create more reliable and validated questionnaires for variables measured by a single item in the present study.

## 5 Conclusion

In alignment with the collective activity hypothesis (8), the current study demonstrated that the resource availability, emphasis on individual performance, and intra-team competition were closely associated with self-enhancement motivation. These results contribute to the assertion that the environment in which the athletes are immersed systematically shapes their motivation, and the effect of past team environments was no exception. Future research should further investigate the effect of socio-ecological factors on athletes' psychological processes to identify nuanced variations among various athletes and to better understand they attempt to enhance their performance.

## Data Availability

The datasets presented in this study can be found in online repositories. The names of the repository/repositories and accession number(s) can be found below: https://osf.io/xr2wz/files/osfstorage/6a268db0285bb438a6980f92.

## Footnotes

^1^We calculated our required sample size based on RMSEA, and the current study met the requirement. Nevertheless, we acknowledge that for the complex model, more data may be required (29).
